# T cell reactivity to the SARS-CoV-2 Omicron variant is preserved in most but not all individuals

**DOI:** 10.1016/j.cell.2022.01.029

**Published:** 2022-03-17

**Authors:** Vivek Naranbhai, Anusha Nathan, Clarety Kaseke, Cristhian Berrios, Ashok Khatri, Shawn Choi, Matthew A. Getz, Rhoda Tano-Menka, Onosereme Ofoman, Alton Gayton, Fernando Senjobe, Zezhou Zhao, Kerri J. St Denis, Evan C. Lam, Mary Carrington, Wilfredo F. Garcia-Beltran, Alejandro B. Balazs, Bruce D. Walker, A. John Iafrate, Gaurav D. Gaiha

**Affiliations:** 1 Massachusetts General Hospital Cancer Center, Massachusetts General Hospital, Boston, MA 02114, USA; 2 Dana-Farber Cancer Institute, Boston, MA 02215, USA; 3 Center for the AIDS Programme of Research in South Africa, Durban 4001, South Africa; 4 Ragon Institute of MGH, MIT and Harvard, Cambridge, MA 02139, USA; 5 Program in Health Sciences & Technology, Harvard Medical School, Massachusetts Institute of Technology, Boston, MA 02115, USA; 6 Department of Pathology, Massachusetts General Hospital, Boston, MA 02114, USA; 7 Massachusetts General Hospital Endocrine Division and Department of Medicine, Harvard Medical School, Boston, MA 02114, USA; 8 Basic Science Program, Frederick National Laboratory for Cancer Research in the Laboratory of Integrative Cancer Immunology, National Cancer Institute, Bethesda, MD 20892, USA; 9 The Broad Institute, Cambridge, MA 02142, USA; 10 Howard Hughes Medical Institute, Chevy Chase, MD 20815, USA; 11 Institute for Medical Engineering and Science, Department of Biology, Massachusetts Institute of Technology, Cambridge, MA 02139, USA; 12 Division of Gastroenterology, Massachusetts General Hospital, Boston, MA 02114, USA

**Keywords:** COVID-19, SARS-CoV-2, T cell, variants, Omicron, Delta, vaccination, neutralization, epitopes, HLA

## Abstract

The SARS-CoV-2 Omicron variant (B.1.1.529) contains mutations that mediate escape from antibody responses, although the extent to which these substitutions in spike and non-spike proteins affect T cell recognition is unknown. In this study, we show that T cell responses in individuals with prior infection, vaccination, both prior infection and vaccination, and boosted vaccination are largely preserved to Omicron spike and non-spike proteins. However, we also identify a subset of individuals (∼21%) with a >50% reduction in T cell reactivity to the Omicron spike. Evaluation of functional CD4^+^ and CD8^+^ memory T cell responses confirmed these findings and revealed that reduced recognition to Omicron spike is primarily observed within the CD8^+^ T cell compartment potentially due to escape from HLA binding. Booster vaccination enhanced T cell responses to Omicron spike. In contrast to neutralizing immunity, these findings suggest preservation of T cell responses to the Omicron variant, although with reduced reactivity in some individuals.

## Highlights

• T cell responses to Omicron are preserved in most infected and vaccinated individuals
• T cell reactivity to Omicron spike is reduced by >50% in ∼20% of individuals
• Most Omicron epitopes have conserved sequence and retain binding to HLA-I
• T cell reactivity to Omicron is enhanced shortly after booster vaccination

## Graphical abstract

**Figure:**
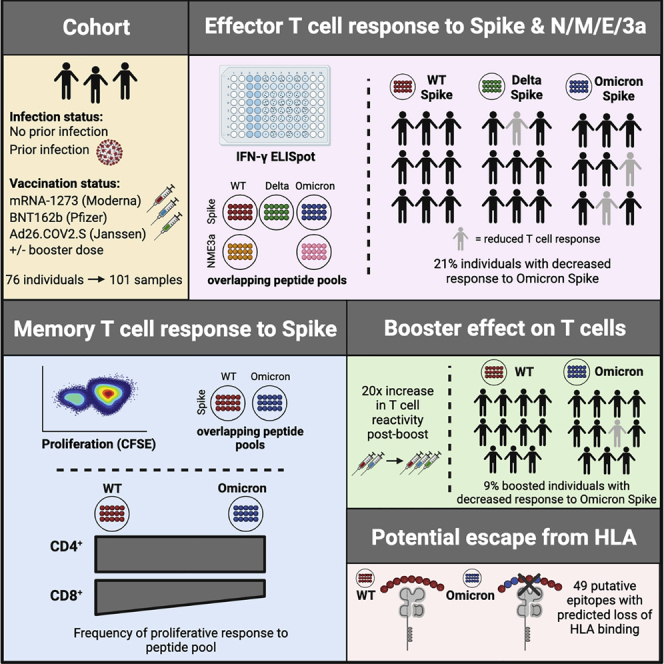

## Teaser

T cells from most SARS-CoV-2 infected and/or vaccinated individuals have preserved reactivity to the Omicron variant, and their reactivity is enhanced by booster vaccination. However, ∼20% of individuals have reduced T cell recognition of the Omicron spike, potentially due to escape of HLA class I-restricted epitopes.

## Introduction

The severe acute respiratory syndrome coronavirus 2 (SARS-CoV-2) Omicron variant (B.1.1.529), first identified in November 2021, has been the cause of a new surge of infections globally (Viana et al., 2021). With as many as 36 substitutions in the viral spike protein and 59 mutations in total throughout its genome, Omicron has been found to evade neutralization by infection- and vaccine-induced antibodies with unprecedented frequency (Garcia-Beltran et al., 2021a; Hoffmann et al., 2021) and escape neutralization by most therapeutic monoclonal antibodies (Ikemura et al., 2021; VanBlargan et al., 2021). Additional booster vaccine doses partially compensate for this effect (Garcia-Beltran et al., 2021b; Hoffmann et al., 2021), but the durability of such protective antibody response remains to be determined. Thus, whether additional arms of the adaptive immune response, namely T cell responses, can augment protection against Omicron infection and disease are of considerable interest and have implications for predicting the course of future SARS-CoV-2 variants.

In individuals with previous SARS-CoV-2 infection and vaccinees, robust T cell responses are quantitatively and qualitatively associated with milder outcomes (Moderbacher et al., 2020). Early induction of antigen-specific CD4^+^ T cells following vaccination is associated with coordinated generation of antibody and CD8^+^ T cell responses (Painter et al., 2021). Previous studies have also shown a key role for CD8^+^ T cells in mitigating COVID-19 disease severity and inducing long-term immune protection. Mild COVID-19 disease is associated with increased clonal expansion of CD8^+^ T cells in bronchoalveloar lavage fluid (Liao et al., 2020), robust CD8^+^ T cell reactivity to SARS-CoV-2 epitopes (Peng et al., 2020; Sekine et al., 2020), and rapid CD8^+^ T cell-mediated viral clearance (Tan et al., 2021). In addition, depletion of CD8^+^ T cells from convalescent macaques reduced protective immunity (McMahan et al., 2021). Given that T cells can target regions across the SARS-CoV-2 proteome and are not limited solely to the spike protein, it is perhaps not unexpected that prior SARS-CoV-2 variants were able to escape neutralizing antibody responses (Garcia-Beltran et al., 2021a), but not T cells (Geers et al., 2021). Thus, in light of the emergence of the Omicron variant, we sought to determine the extent to which mutations in the variant spike and nonspike proteins affect CD4^+^ and CD8^+^ T cell reactivities.

Utilizing samples from prior SARS-CoV-2 infected, vaccinated, and both prior infected and vaccinated individuals, we found that circulating effector T cell responses and both CD4^+^ and CD8^+^ memory T cell responses were generally preserved to the Omicron variant. However, distinct from previous variants of concern (VOCs), such as Delta, a subset of individuals had reduced effector and memory T cell recognition to the Omicron spike protein relative to wild-type spike, with a particularly noticeable effect on spike-specific CD8^+^ T cell memory responses. Booster doses enhanced the magnitude of responses to wild-type and Omicron spike, although did not completely mitigate the comparatively reduced T cell reactivity to Omicron in individual participants. These findings therefore have important implications in ascertaining the role of immune responses in morbidity and mortality due to Omicron and may inform the development of variant-specific and variant-resistant second-generation vaccines.

## Results

**Figure 1:**
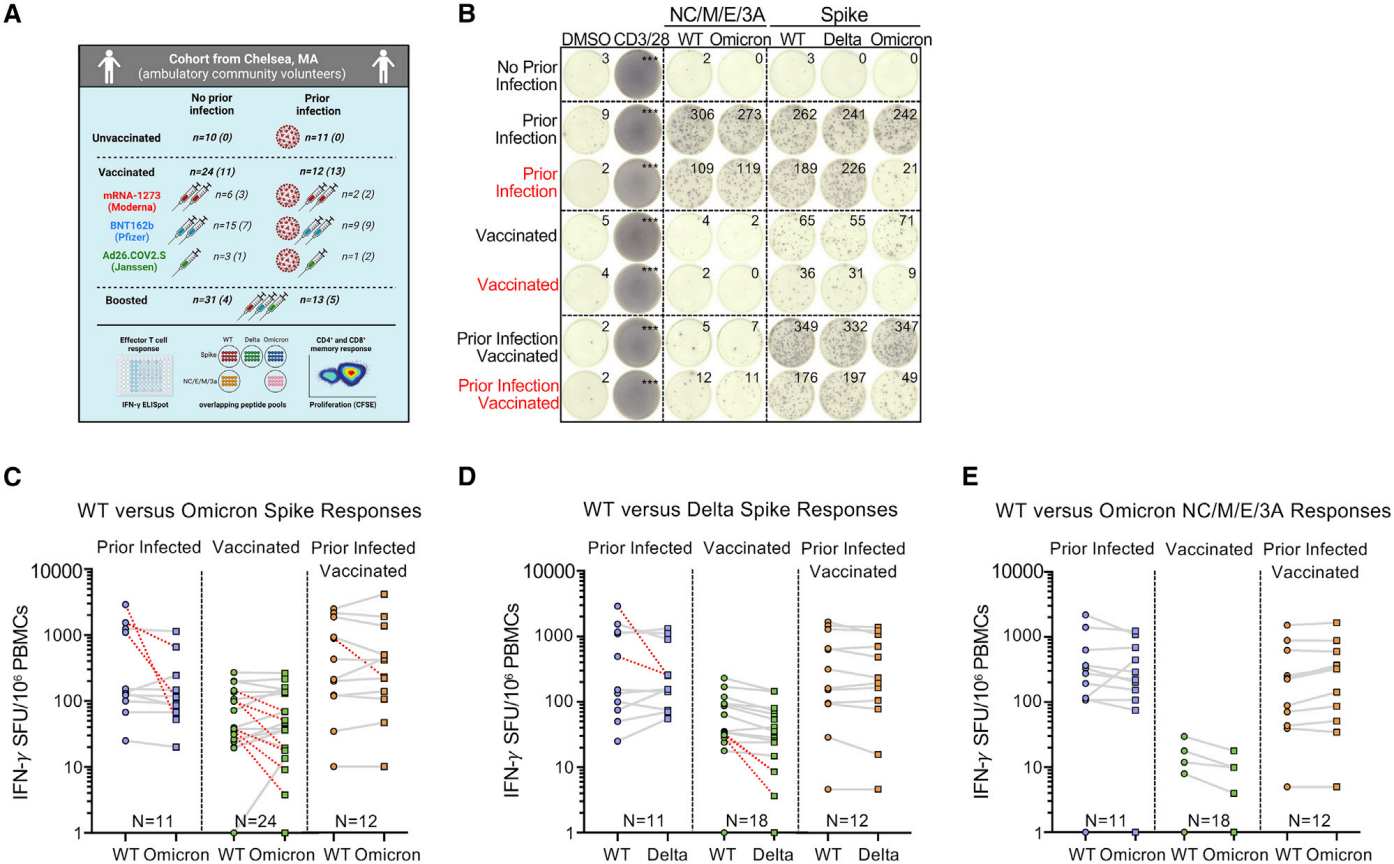
Effector T cell reactivity to the SARS-CoV-2 Omicron variant is preserved in most but not all individuals with prior infection and/or primary series vaccination (A) Schematic of the study created with Biorender.com: participants were enrolled in Chelsea, Massachusetts and were stratified according to whether they had documented asymptomatic or symptomatic SARS-CoV-2 infection (ascertained by antinucleocapsid antibody testing) and vaccination status (see Table S1). In total, 101 PBMC samples from 76 individuals were studied. However, 25 individuals provided samples prior to and after receipt of additional booster vaccine doses. Total (CD4^+^ and CD8^+^) effector T cell reactivity to SARS-CoV-2 overlapping peptide pools from wild-type, Omicron, or Delta spike and from wild-type or Omicron nonspike structural and accessory proteins (nucleocapsid/membrane/envelope/ORF3A, i.e., NC/M/E/3A) was assessed by IFN-γ ELISpot (the number is shown for each group). CD4^+^ and CD8^+^ memory T cell response to wild-type or Omicron spike was assessed in a subset of participants by CFSE-based proliferation assay (see Figure 3). Numbers for each group are shown in parentheses. Table S2 describes the degree of overlap in peptide pools. (B) Representative IFN-γ ELISpot responses for study participants following no stimulation (dimethyl sulfoxide [DMSO] only), anti-CD3 and anti-CD28 stimulation (positive control), overlapping NC/M/E/3A peptide pools from wild-type SARS-CoV-2 and Omicron variant, and overlapping spike peptide pools from wild-type, Delta, and Omicron variant. Those delineated in red are representative examples of individuals with >50% decreased T cell responses to the Omicron spike peptide pool compared with wild type. The median magnitude of wild-type or Omicron spike responses among negative controls (individuals without prior infection or vaccination) was 0–5 SFU/10^6^ cells (see Figure S1). (C) Comparative IFN-γ ELISpot spot forming units (SFUs) per 10^6^ peripheral blood mononuclear cells (PBMCs) in individuals with prior infection, vaccination, and both prior infection and vaccination with overlapping wild-type and Omicron spike peptide pools. Overall T cell responses to wild type and Omicron were comparable across all groups by multivariate regression, although red dashed lines indicate the 10 participants with a >50% decrease (0.3log_10_) in reactivity. (D) Comparative IFN-γ ELISpot responses in individuals with prior infection, vaccination, and both prior infection and vaccination with overlapping wild-type and Delta spike peptide pools. Overall T cell responses to wild type and Delta were comparable across all groups, although red dashed lines indicate the 4 participants with a >50% decrease (0.3log_10_) in reactivity. (E) Comparative IFN-γ ELISpot responses in individuals with prior infection, initial vaccination series, and both prior infection and vaccination to overlapping peptide pools of the wild type and Omicron NC/M/E/3A. In (C–E), each dot is a single participant. Circles denote responses to wild-type peptides and squares to Omicron or Delta peptides. Dots are colored by prior infection and vaccine stratum (blue for prior infection and no vaccination, green for no prior infection and vaccination with primary series, and orange for both prior infection and vaccination with primary series). In (C–E), pair-wise comparison of effector T cell reactivity toward wild type versus variant by a paired t test (not adjusted for multiple comparisons or covariates) was not significant. See also Figures S1 and S2 and Tables S1–S4.

To assess the cross-reactivity of T cell responses to the Omicron variant, we studied anti-SARS-CoV-2 T cell responses in 76 ambulatory adult volunteers in Chelsea, Massachusetts sampled prior to vaccination, after primary series vaccination, and/or after receipt of additional “booster” doses. Study groups were stratified by prior infection (confirmed by anti-nucleocapsid antibody testing) and vaccination status (Table S1). In total, we studied 101 samples from 76 donors (Figure 1A). The median age was 45 years (range 37–60 years), and 64% were female. Of the previously infected individuals, we included 11 unvaccinated, 12 vaccinated sampled after initial vaccination series, and 13 vaccinated and sampled after booster doses individuals. Among individuals without previous infection, we included 10 unvaccinated, 24 sampled after initial vaccination series, and 31 vaccinated and sampled after booster doses individuals. Samples were obtained at a median 220 (range 130–286) days after primary series vaccination or 10 days (range 8–54) after additional booster doses. The primary analysis of host, vaccine, and variant variables that affect T cell responses was by multivariate regression.

### Effector T cell reactivity to the SARS-CoV-2 Omicron variant is preserved in most but not all prior infected and vaccinated individuals

To assess the total (CD4^+^ and CD8^+^) effector T cell response, we performed an IFN-γ ELISpot following stimulation with pooled overlapping 15-mer peptides spanning the full length of the wild-type, Delta (B.1.617.2), or Omicron (B.1.1.529) spike protein and the nonspike SARS-CoV-2 structural and accessory proteins (nucleocapsid, membrane, enveloped, and open reading frame 3A, i.e., NC/M/E/3A) from wild type and Omicron. The evaluated peptides span the full-length of spike: relative to wild type, 27.3% (86/315) spike peptides were unique to Omicron and 8.6% (27/315) to Delta. For the NC/M/E/3A pools, the evaluated peptides span the full-length of the respective proteins: relative to wild type, 10.1% (24/237) of the peptides were unique to Omicron (Table S2). In the primary multivariate analysis of T cell reactivity (Table S3), the magnitude of effector T cell responses to spike and nonspike proteins did not vary by variant and was not affected by age, sex, and primary vaccine series. However, examination of individual responses reveals specific patients in whom responses to the Omicron spike were reduced by >50% (Figure 1B, denoted in red). Prior infection, duration after primary series vaccination, and receipt of an additional “booster” dose were independently associated with magnitude of response (Table S3).

**Figure S1:**
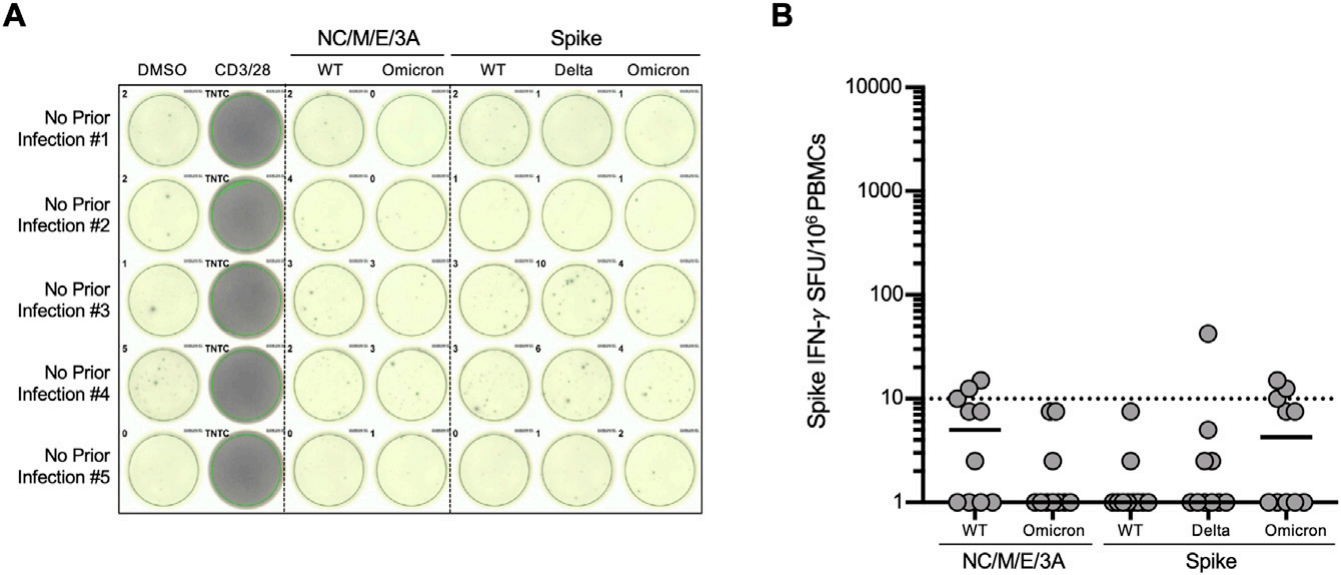
The magnitude of wild-type or Omicron effector T cell responses in negative individuals without prior infection or vaccination, related to Figure 1 (A) Representative IFN-γ ELISpot responses for five participants with no prior SARS-CoV-2 infection (by history and confirmed with negative antinucleocapsid antibody testing), following no stimulation (dimethyl sulfoxide [DMSO] only), anti-CD3 and anti-CD28 stimulation (positive control), overlapping NC/M/E/3A peptide pools from wild-type SARS-CoV-2 and Omicron variant, and overlapping spike peptide pools from wild-type, Delta and Omicron variant. (B) The magnitude of wild-type or omicron nucleocapsid, membrane, envelope, ORF3A (NC/M/3A), and spike in ten evaluated participants. For each peptide pool, the horizontal line denotes the median. The horizontal dotted line denotes a response of 10 SFU/10^6^ PBMC.

The background response to wild-type, Delta, or Omicron peptide pools in individuals without prior SARS-CoV-2 infection or vaccination had median values of 0–5 SFU/10^6^ peripheral blood mononuclear cells (PBMCs) (Figure S1). The median effector T cell reactivity against wild-type and Omicron spike (in SFU/10^6^ PBMC) was 152 and 114 for individuals with prior infection, 43 and 42 for individuals after primary series vaccination (without prior infection), and 311 and 315 for individuals with prior infection after primary series vaccination (Figure 1C). In comparison, the median effector T cell reactivity against Delta spike (in SFU/10^6^ PBMC) was 155 for individuals with prior infection, 34 for individuals after primary series vaccination (without prior infection), and 277 for individuals with prior infection after primary series vaccination (Figure 1D). Regardless of variant, prior infection was associated with a higher magnitude of effector T cell responses (0.55 log_10_ SFU/10^6^ PBMC higher response, 95% CI 0.38–0.72, p < 0.001). Effector T cell responses declined modestly over time (−0.02 log_10_ SFU/10^6^ PBMC lower response per week, 95% CI −0.05, 0.00, p = 0.028). Neither age nor sex influenced responses, and in this analysis, primary vaccine type was not associated with differences in responses (Table S3). Surprisingly, 21.2% (10/47) of participants with prior infection and/or vaccination had a >50% (0.3log_10_) reduction in T cell response to Omicron spike (denoted in red in Figure 1C), with 12.7% of participants (6/47) having a >70% (0.5 log_10_) reduction. In contrast, only 9.7% (4/41) of participants with prior infection and/or vaccination had a >50% (0.3log_10_) reduction in overall effector T cell response to Delta spike. Thus, while T cell responses induced by prior infection and/or vaccination are broadly cross-reactive at a population level, a distinct subset of individuals have substantially reduced T cell recognition of the mutated Omicron spike protein.

**Figure S2:**
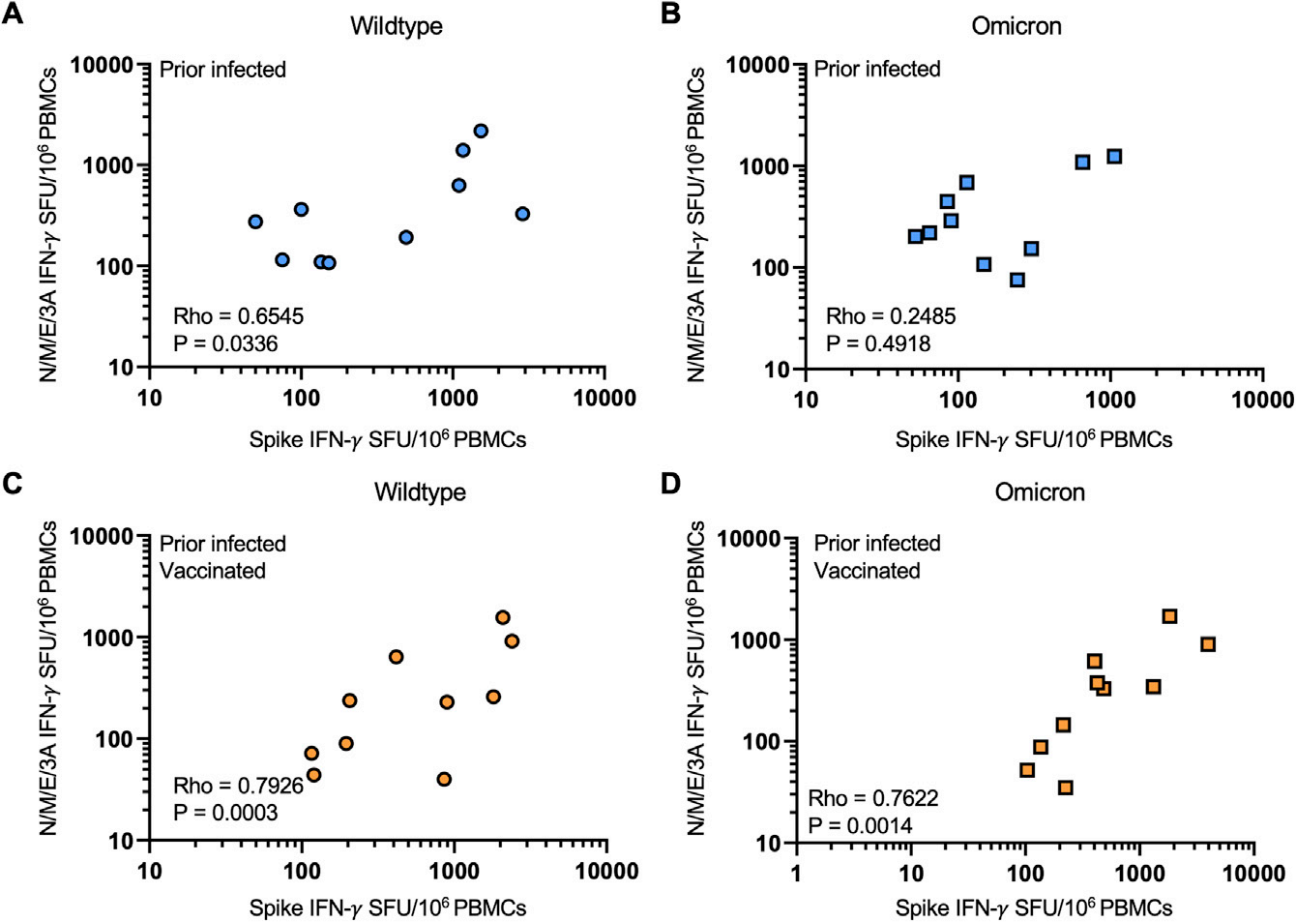
Effector T cell responses to spike and nonspike structural proteins are correlated for wild type and Omicron among individuals with prior infection with or without vaccination, related to Figure 1 (A) Scatter plot of magnitude of effector T cell response (IFN-γ SFU per 10^6^ PBMCs) directed against spike and nonspike structural and accessory proteins (nucleocapsid, membrane, envelope, and ORF3A) from wild-type SARS-CoV-2 in prior infected individuals. (B) Scatter plot of magnitude of effector T cell response (IFN-γ SFU per 10^6^ PBMCs) directed against spike and nonspike structural and accessory proteins (nucleocapsid, membrane, envelope, and ORF3A) from the SARS-CoV-2 Omicron variant in prior infected individuals. (C) Scatter plot of magnitude of effector T cell response (IFN-γ SFU per 10^6^ PBMCs) directed against spike and nonspike structural and accessory proteins (nucleocapsid, membrane, envelope, and ORF3A) from wild-type SARS-CoV-2 in prior infected and vaccinated individuals. (D) Scatter plot of magnitude of effector T cell response (IFN-γ SFU per 10^6^ PBMCs) directed against spike and nonspike structural and accessory proteins (nucleocapsid, membrane, envelope, and ORF3A) from the SARS-CoV-2 Omicron variant in prior infected and vaccinated individuals. In (A–D), each dot is a single participant. Circles denote responses to wild-type peptides and squares to Omicron peptides. Dots are colored by prior infection and vaccine stratum (blue prior infection and no vaccination and orange for both prior infection and vaccination with primary series). Spearman correlation coefficients are denoted in each panel.

In contrast to spike-specific T cell responses, T cell reactivity against wild-type and Omicron NC/M/E/3A was preserved in all individuals with prior infection (with or without subsequent vaccination). The median effector T cell reactivity against wild-type and Omicron NC/M/E/3A (in SFU/10^6^ PBMC) was 275 and 220 for individuals with prior infection, 1 and 0 for individuals after primary series vaccination (without prior infection), and 160 and 237 for individuals with prior infection and after primary series vaccination (Figure 1E). In individuals with prior infection or prior infection and vaccination, the magnitude of reactivity toward NC/M/E/3A was correlated with that of spike for wild-type and Omicron peptides (Figure S2).

### Additional booster vaccine doses enhance effector T cell responses to SARS-CoV-2 wild type and Omicron

**Figure 2:**
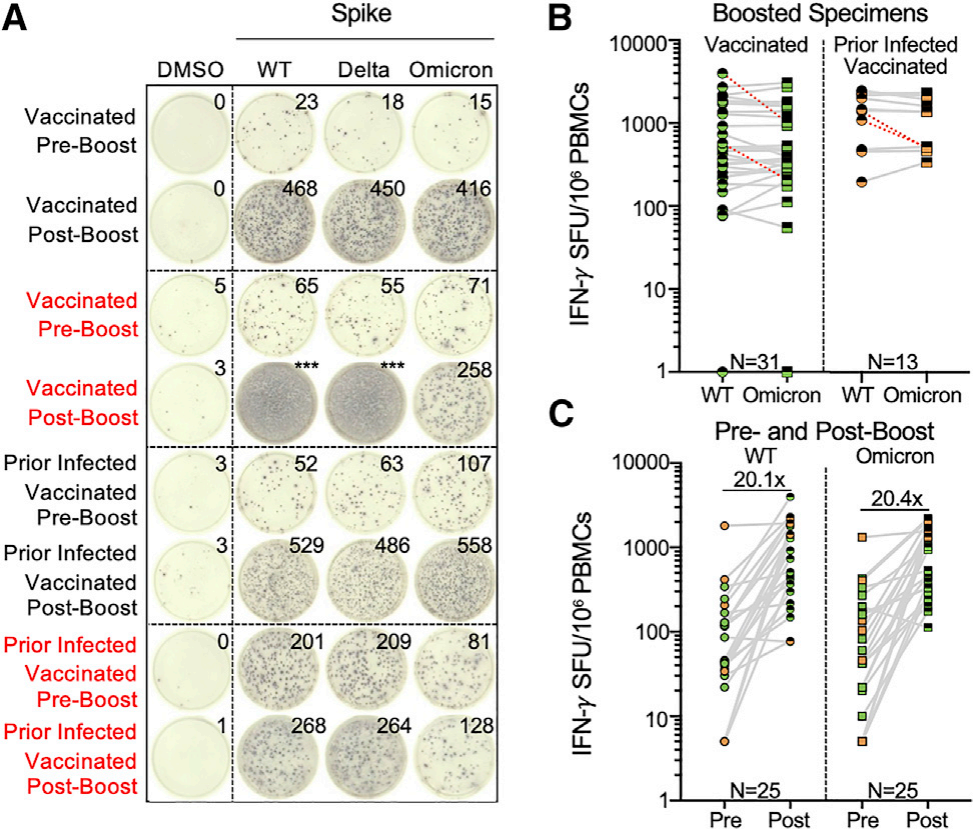
Effector T cell responses to SARS-CoV-2 wild type and Omicron are enhanced by additional booster vaccination (A) Representative IFN-γ ELISpot responses for study participants. Shown are IFN-γ ELISpot responses following no stimulation (dimethyl sulfoxide [DMSO] only), anti-CD3 and anti-CD28 stimulation (positive control), and overlapping spike protein peptide pools from wild-type and Omicron variant in individuals with no prior infection and vaccinated or with prior infection and vaccinated sampled prior to receipt of booster (preboost) or after booster (postboost) vaccine doses. Those delineated in red indicate representative examples of individuals with >50% decreased T cell responses to the Omicron spike peptide pool in comparison with wild type. (B) Comparative IFN-γ ELISpot responses in individuals with and without prior infection following booster vaccination (range 8–54 days following booster dose). Red dashed lines indicate the 4 participants with a >50% decrease (0.3log_10_) in T cell reactivity to Omicron relative to wild type. (C) Comparative T cell reactivity pre and postbooster vaccination (8–10 days following booster dose) in 25 participants to both wild-type and Omicron spike protein. Booster vaccination elicited an ∼20-fold increase in T cell response magnitude to both spike proteins. In (B and C), each dot is a single participant. Circles denote responses to wild-type peptides and squares to Omicron peptides. Dots are colored by prior infection and vaccine stratum (green for no prior infection and vaccination with primary series and orange for both prior infection and vaccination with primary series). Fill denotes sampling preboost (full filled) or postboost (half filled). In (B), pair-wise comparison of effector T cell reactivity toward wild type versus variant by a paired t test (not adjusted for multiple comparisons or covariates) was not significant. See also Figure 2 and Tables S3 and S4.

Individuals with prior infection demonstrated higher T cell responses to spike, suggesting that repeated exposure to antigen may potentially enhance cross-reactive T cell responses. We therefore assessed the impact of booster vaccination on T cell reactivity by IFN-γ ELISpot. Similar to the evaluation of preboost samples, overall effector T cell responses toward wild-type and Omicron spike across our study participants were comparable with postbooster (Figure 2B). Moreover, receipt of a booster dose was associated with a 1.1log_10_ SFU/10^6^ PBMC increase (95% CI 0.91–1.2, p < 0.001) in the magnitude of T cell response (Table S3), with specific fold increases of 20.1 against wild type and 20.4 against Omicron in 25 participants with paired sampling (Figure 2C). However, even after booster vaccination, 9.1% (4/44) participants still demonstrated >50% reduced reactivity to Omicron spike relative to wild type. Overall, the frequency of >50% reduced effector T cell responses to the Omicron variant was more frequent than to Delta in 85 individual sample points with both measures (Fisher’s exact p value 0.023, Table S4).

### Proliferative CD4^+^ memory T cell responses are preserved against Omicron but CD8^+^ T cell responses are reduced

**Figure S3:**
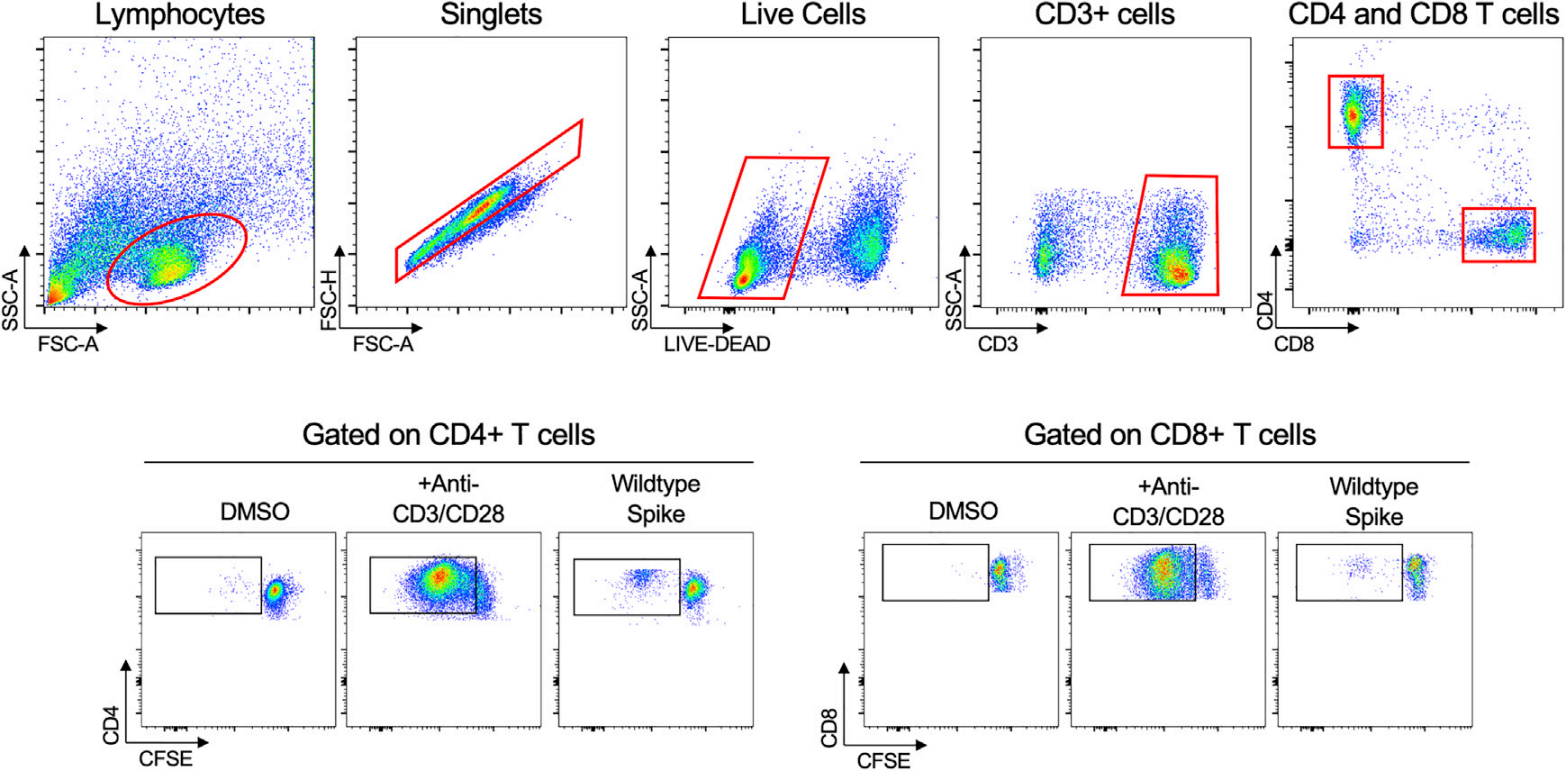
Gating strategy for CFSE proliferation assay, related to Figure 3 Representative gating strategy for identification of proliferating CD3^+^ CD4^+^ and CD3^+^ CD8^+^ CFSE low T cells in response to peptide pools of interest. The gate establishing the frequency of CFSE low CD4 ^+^ or CD8 ^+^ cells was chosen based on minimizing responses in two negative-control (DMSO) wells and verified using positive control (CD3/CD28) wells.

**Figure 3:**
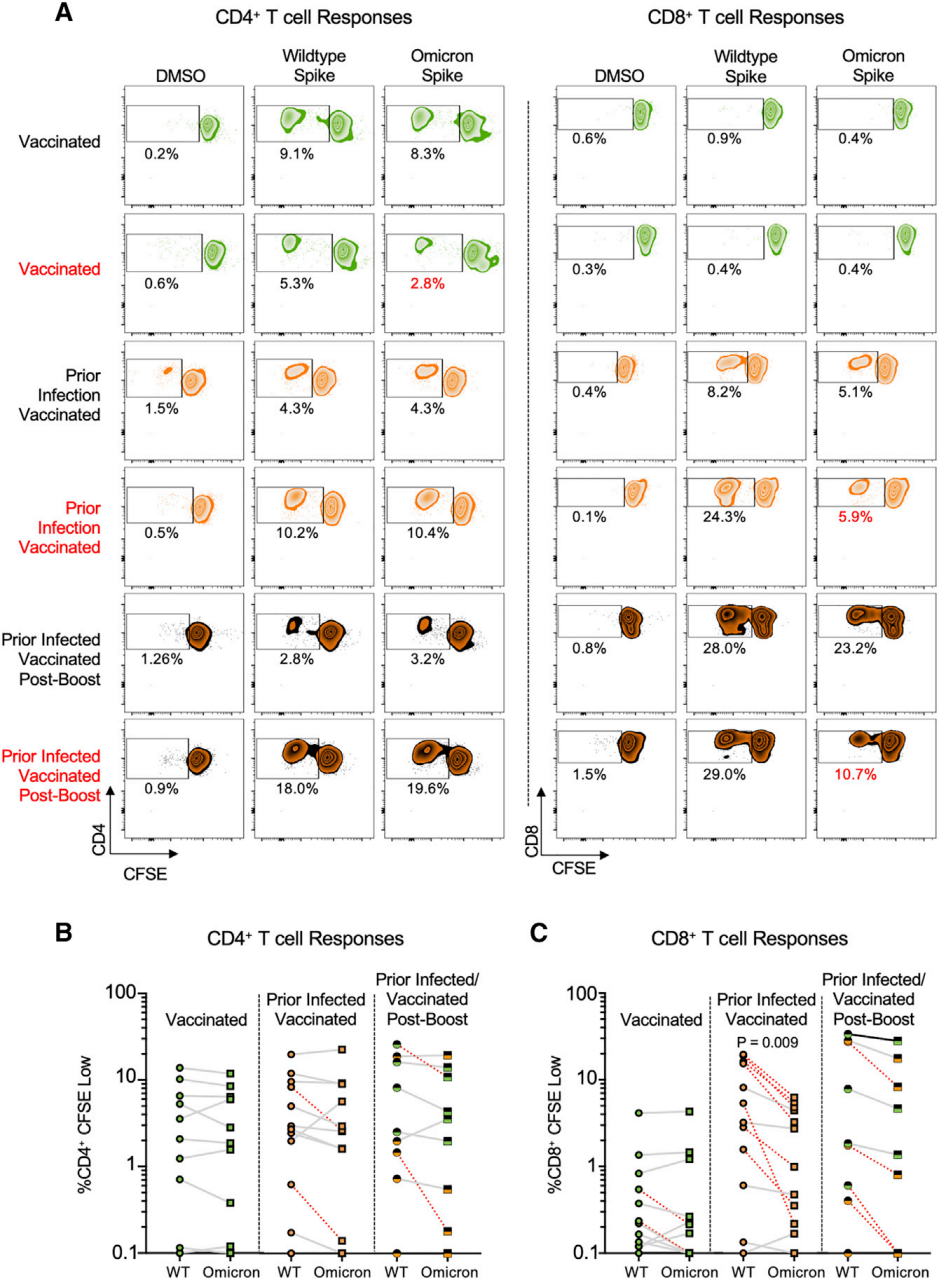
Proliferative spike-specific CD4^+^ T cell responses are preserved against Omicron, but CD8^+^ T cell responses are reduced (A) Representative CFSE responses for study participants. Shown are CD4^+^ (left) and CD8^+^ (right) T cell responses following no stimulation (dimethyl sulfoxide [DMSO] only) and overlapping spike protein peptide pools from wild-type and Omicron variant. Those delineated in red indicate representative examples of individuals with >50% decreased T cell responses to the Omicron spike peptide pool in comparison with wild type. (B) Comparative %CD4^+^ CFSE Low cells in individuals with vaccination, both prior infection and vaccination, and prior infection, initial vaccination, and booster vaccination in response to overlapping wild-type and Omicron spike peptide pools. Data are means of technical duplicates. Red dashed lines indicate the 4 individuals with a >50% decrease (0.3log_10_) in proliferative response. Pair-wise comparison of memory CD4^+^ T cell proliferation to wild-type versus Omicron spike by a paired t test (not adjusted for multiple comparisons or covariates) was not significant. (C) Comparative %CD8^+^ CFSE Low cells in individuals with vaccination, both prior infection and vaccination, and prior infection, initial vaccination, and booster vaccination in response to overlapping wild-type and Omicron spike peptide pools. Data are means of technical duplicates. Red dashed lines indicate the 13 individuals with a >50% decrease (0.3log_10_) in proliferative response. Pair-wise comparison of proliferative CD8^+^ T cell responses in previously infected, vaccinated individuals (p = 0.009) and across all study participants (p < 0.005) to wild-type versus Omicron spike by a paired t test (not adjusted for multiple comparisons or covariates) revealed a significant reduction in response to Omicron spike. See Figure S3 and Table S5.

To assess the cross-reactivity of functional CD4^+^ and CD8^+^ memory T cell responses to Omicron, we performed a 6-day carboxyfluorescein succinimidyl ester (CFSE) proliferation assay (Figure S3) on samples from individuals who were vaccinated and/or previously infected and/or received booster vaccine doses (n = 33 participants) using overlapping wild-type or Omicron spike peptide pools. We felt it was important to utilize this assay, given that antigen-specific proliferation has been strongly associated with functional T cell responses and cytotoxicity (Migueles et al., 2002, 2008; Ndhlovu et al., 2013). The patient cohort studied here was a subset of that used for the IFN-γ ELISpot assay, wherein 11 samples were from vaccinated individuals, 13 from previously infected and vaccinated individuals, and nine from vaccinated and boosted individuals (five of whom were also previously infected). A paired t test demonstrated that although the magnitude of proliferative spike-specific CD4^+^ T cell responses did not vary by variant, proliferative CD8^+^ T cell responses to Omicron spike were decreased compared with wild type in previously infected, vaccinated participants (p = 0.009), and across all study participants (p < 0.005), which is further illustrated by examination of individual patient responses (Figure 3A, denoted in red). CD4^+^ proliferative responses remained largely cross-reactive to Omicron spike, with only 12% (4/33) of individuals with prior infection and/or vaccination and/or booster showing a >50% (0.3log_10_) reduction (Figure 3B). A larger proportion of 39% of individuals (13/33) exhibited a decreased CD8^+^ T cell proliferative response to Omicron spike (Figure 3C). A multivariate regression analysis revealed that neither age nor sex influenced CD4^+^ or CD8^+^ T cell responses, but proliferative CD8^+^ responses tended to be lower for Omicron versus wild type after adjusting for all covariates and were significantly increased by booster doses (Table S5). These data indicate that the reduced reactivity in a subset of individuals to the Omicron spike protein is primarily observed in the CD8^+^ T cell compartment, although this can be enhanced with booster vaccination.

### Identification of putative spike CD8^+^ T cell epitopes with a predicted loss of HLA binding due to mutations in the Omicron variant

To determine whether mutations in the Omicron spike protein could affect recognition of HLA class I-restricted CD8^+^ T cell epitopes, we computationally assessed the predicted binding affinity of all 8- to 11-mer peptides in the wild-type (N = 5,058) and Omicron spike (N = 5,050) for 150 HLA-A, -B, and -C alleles using NetMHCpan4.1 (Reynisson et al., 2020). We chose to evaluate this large group of HLA alleles in order to be as comprehensive as possible, given the global nature of the pandemic. This identified 736 unique epitopes that were predicted to be strong binders to HLA class I molecules (Table S6). The majority of these epitopes (83.2%, 612/736) were identical in sequence between wild type and Omicron, and of the 16.8% (124/736) epitopes that had sequence differences, 60.4% (75/124) retained similar predicted HLA class I binding affinity. However, for the remaining epitopes with sequence differences (6.7%, 49/736), the Omicron spike mutations resulted in a predicted loss of binding to one or more HLA alleles (Table 1). Thus, the analysis provides a possible explanation as to why the mutations in the Omicron spike protein could lead to a decrease in epitope presentation and subsequently T cell reactivity.

**Table 1:** CD8^+^ T cell epitopes in the SARS-CoV-2 spike protein with a reduction in predicted binding affinity due to mutations in Omicron

WT epitope	Omicron epitope^a^	Position	HLA allele^b^	WT epitope EL_rank	Omicron epitope EL_rank^c^
VTWFHAIHV	VTWFH**V**I**SG**	62–70	HLA-A02:06, HLA-A68:02, HLA-A68:31, HLA-A69:01, **HLA-C15:02**, HLA-C15:05, HLA-C15:13	0.0994–0.4749	14.7857–20.8995
FHAIHVSGT	FH**V**I**SG**TNG	65–73	HLA-B39:06	0.3037	3.2558
HVSGTNGTK	**VI**SGTNGTK	69–77	HLA-A66:01, **HLA-A68:01**, HLA-A68:24	0.3145–0.4865	2.1231–2.6776
HVSGTNGTKR	**VI**SGTNGTKR	69–78	HLA-A66:01	0.4918	4.3511
STEKSNIIRGW	S**I**EKSNIIRGW	94–104	HLA-B57:03	0.3307	2.1519
FQFCNDPFLGV	FQFCNDPFL**DH**	133–143	HLA-A02:05, HLA-A02:06	0.1472–0.271	11.2109–16.5747
QFCNDPFLGVY	QFCNDPFL**DHK**	134–144	**HLA-A29:02**	0.4522	11.7412
FCNDPFLGV	FCNDPFL**DH**	135–143	HLA-A02:06, HLA-A69:01, **HLA-C15:02**, HLA-C15:13	0.3404–0.4963	12.3627–38.0909
FCNDPFLGVY	FCNDPFL**DHK**	135–144	**HLA-A01:01**, HLA-B46:01, HLA-C03:02	0.3813–0.4899	4.6195–20.2193
CNDPFLGVY	CNDPFL**DHK**	136–144	**HLA-A01:01**	0.3936	4.7481
CNDPFLGVYY	CNDPFL**DHKN**	136–145	**HLA-A01:01**	0.3849	46
DPFLGVYY	DPFL**DHKN**	138–145	**HLA-B35:01**, HLA-B35:05, HLA-B35:17	0.327–0.3615	16.9813–17.8876
GVYYHKNNK	**DHKNN**K**SWM**	142–150	HLA-A03:01, HLA-A03:02, **HLA-A11:01, HLA-A30:01**, HLA-A34:02, HLA-A68:30, HLA-A74:01, HLA-A74:03	0.0456–0.4975	30.1368–73.8889
VYYHKNNKSW	**FLD**HKNNKSW	143–152	**HLA-A23:01, HLA-A24:02**, HLA-A24:03	0.1995–0.2308	2.9545–3.9173
YYHKNNKSW	**LD**HKNNKSW	144–152	**HLA-A23:01, HLA-A24:02**, HLA-A24:03, HLA-A24:14, **HLA-C07:02**, HLA-C07:17, HLA-C14:02	0.0735–0.2886	5.9289–28.0526
YYHKNNKSWM	**LD**HKNNKSWM	144–153	HLA-C14:02	0.2442	18.2877
IYSKHTPINL	IYSKHTPI**IV**	203–212	**HLA-A23:01**, HLA-A24:14	0.3433–0.3775	2.3069–2.6038
YSKHTPINL	YSKHTPI**IV**	204–212	HLA-B15:17, **HLA-C01:02**	0.3669–0.3895	2.0205–2.3964
SKHTPINLV	SKHTPI**IVE**	205–213	**HLA-B52:01, HLA-C06:02**	0.222–0.4776	15.4455–21.7107
KHTPINLVRDL	KHTPI**IVEPE**R	206–216	**HLA-B38:01**	0.3389	14.8824
TPINLVRDL	TPI**IVEPER**	208–216	HLA-B07:02, HLA-B07:05, HLA-B35:02, HLA-B35:03, HLA-B35:04, HLA-B35:05, HLA-B35:09, HLA-B35:12, HLA-B35:17, HLA-B39:10, HLA-B40:08, HLA-B42:01, **HLA-B42:02**, HLA-B78:01	0.0931–0.3426	2.2247–7.3816
LVRDLPQGF	**IVEPE**RD**LP**	212–220	HLA-A25:01, **HLA-A26:01**, HLA-A26:03, HLA-A26:08, HLA-A26:27, **HLA-A32:01, HLA-B15:01**, HLA-B15:16, HLA-B15:17, **HLA-B15:20**, HLA-B15:71, HLA-B35:43, HLA-B46:01, **HLA-C02:02**, HLA-C02:10, HLA-C03:02, **HLA-C12:02, HLA-C15:09**	0.1169–0.4993	28.5161–57.2222
VRDLPQGF	V**EPE**RDLP	213–220	HLA-C18:02, HLA-C18:11	0.3921	51.25
VRDLPQGFSA	V**EPE**RDLPQG	213–222	HLA-B39:06	0.4636	62.5
VRDLPQGFSAL	V**EPE**RDLPQGF	213–223	**HLA-C01:02**	0.1845	19.9423
GEVFNATRF	**D**EVFNATRF	339–347	HLA-B15:71, **HLA-B40:02**	0.3426–0.4738	2.0478–2.1667
GEVFNATRFA	**D**EVFNATRFA	339–348	HLA-B40:06	0.2498	2.2154
SASFSTFKCY	**L**A**P**F**F**TFKCY	371–380	HLA-A30:10	0.4334	3.875
ASFSTFKCY	A**P**F**F**TFKCY	372–380	**HLA-A30:02**, HLA-A30:04, HLA-A30:10, **HLA-B15:01**, HLA-B15:03, HLA-B15:16, HLA-B15:17, HLA-B15:220, HLA-B15:71, HLA-B46:01, HLA-B58:02, **HLA-C02:02**, HLA-C02:10, **HLA-C12:02, HLA-C12:03, HLA-C15:09, HLA-C16:01**, HLA-C16:02, HLA-C16:04	0.0377–0.4525	2.1721–10.8571
RQIAPGQTGK	RQIAPGQTG**N**	408–417	HLA-A03:01, HLA-A03:02, **HLA-A11:01, HLA-A30:01**, HLA-A74:01, HLA-A74:03	0.0396–0.4355	17.9689–31.8333
QIAPGQTGK	QIAPGQTG**N**	409–417	HLA-A03:01, HLA-A03:02, **HLA-A11:01**, HLA-A34:02, HLA-A66:01, **HLA-A68:01**, HLA-A68:24, HLA-A68:30	0.054–0.4149	14.1713–30.5
KIADYNYKL	**N**IADYNYKL	417–425	HLA-A30:04, **HLA-A32:01**, HLA-B13:02	0.0751–0.4474	2.3954–2.9282
QSYGFQPTNGV	**R**SY**S**F**R**PT**Y**GV	493–503	HLA-A68:02	0.3745	3.0222
GFQPTNGVGY	**S**F**R**PT**Y**GVG**H**	496–505	**HLA-A29:02, HLA-A30:02**, HLA-A30:04, HLA-B15:71	0.234–0.4406	2.8292–7.2439
FQPTNGVGY	F**R**PT**Y**GVG**H**	497–505	**HLA-B15:01**, HLA-B15:03, **HLA-B15:20**, HLA-B15:220, HLA-B15:71, HLA-B35:20, HLA-B46:01, **HLA-C12:02**	0.0946–0.4381	6.3617–32.0638
VGYQPYRV	VG**H**QPYRV	503–510	**HLA-B52:01**	0.4827	2.8193
GYQPYRVVV	G**H**QPYRVVV	504–512	HLA-C14:02	0.3362	3.1636
GYQPYRVVVL	G**H**QPYRVVVL	504–513	HLA-A24:03	0.4417	7.9017
YQDVNCTEV	YQ**G**VNCTEV	612–620	HLA-A02:18, **HLA-B39:05**, HLA-B39:08, HLA-B39:11, **HLA-C04:01**, HLA-C05:01, HLA-C07:04, HLA-C08:01, HLA-C08:02, HLA-C08:35, HLA-C18:02, HLA-C18:11	0.1391–0.457	2.6799–5.173
EHVNNSYEC	E**Y**VNNSYEC	654–662	HLA-B15:09, HLA-B39:01, **HLA-B39:05**, HLA-B39:06, HLA-B39:31	0.1167–0.4895	4.7388–9.8507
QTNSPRRAR	QT**K**S**H**RRAR	677–685	HLA-A68:30	0.283	2.6055
TNSPRRARSVA	T**K**S**H**RRARSVA	678–688	HLA-B55:01	0.4083	20.2222
NSPRRARSV	**K**S**H**RRARSV	679–687	**HLA-C01:02**	0.2382	2.7537
NSPRRARSVA	**K**S**H**RRARSVA	679–688	HLA-B55:01	0.3381	11.7403
SPRRARSV	S**H**RRARSV	680–687	HLA-B07:02, HLA-B07:05, **HLA-B08:01**, HLA-B42:01, **HLA-B42:02**, HLA-B55:01	0.2012–0.4236	2.2652–10.8585
SPRRARSVA	S**H**RRARSVA	680–688	HLA-B07:02, HLA-B07:05, HLA-B42:01, **HLA-B42:02**, HLA-B55:01, HLA-B55:02, HLA-B56:01, HLA-B78:01	0.0057–0.2316	2.7233–11.1698
SPRRARSVAS	S**H**RRARSVAS	680–689	HLA-B55:01	0.3492	22.505
KDFGGFNF	K**Y**FGGFNF	795–802	HLA-B37:01	0.4013	19.0325
SNFGAISSV	S**K**FGAISSV	968–976	**HLA-C15:02**, HLA-C15:13	0.3611	2.4174

See also Tables S6 and S7.

a Bolded amino acids in the Omicron peptide are those that differ from the wild-type (WT) peptide.

b Bolded HLA class I alleles are those that are present in 10 individuals with >50% decreased reactivity to Omicron spike.

c Published thresholds (Reynisson et al., 2020) were used to define strong binders as those with an EL_rank score <0.5, weak binders as those with scores >0.5 and <2.0 and non-binders as those with scores >2.0.

In addition, we obtained the HLA haplotypes for 10 individuals with a >50% reduction in either their effector or memory T cell responses to the Omicron spike. Interestingly, all of these study participants expressed at least three HLA class I alleles that are well bound by wild-type-derived epitopes, but which are predicted to be nonbinders to corresponding Omicron-derived epitopes (Table S7). Indeed, the majority (60%, 6/10 individuals) carried between 4–6 HLA class I alleles affected by Omicron spike mutations (Table 1), illustrating a putative susceptibility to viral epitope escape.

### Effector T cell responses to Omicron are preserved even in individuals with undetectable neutralization of Omicron

**Figure 4:**
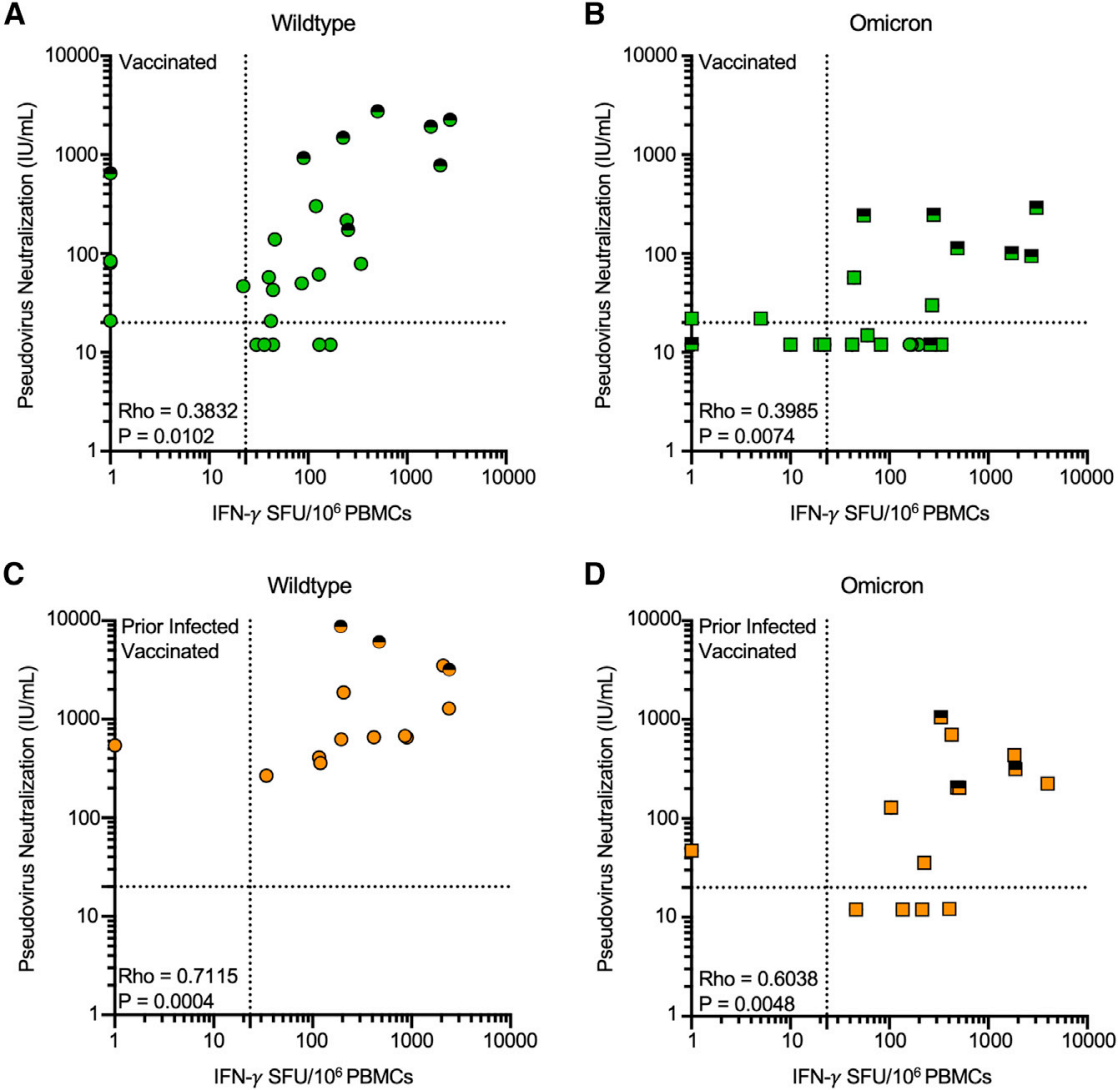
Effector T cell responses to Omicron are present in individuals with undetectable neutralization of Omicron (A) Scatter plot of magnitude of effector T cell response (IFN-γ SFU per 10^6^ PBMCs) and Pseudovirus neutralization (IU/mL) against wild-type SARS-CoV-2 in vaccinated individuals. (B) Scatter plot of magnitude of effector T cell response (IFN-γ SFU per 10^6^ PBMCs) and Pseudovirus neutralization (IU/mL) against SARS-CoV-2 Omicron variant in vaccinated individuals. (C) Scatter plot of magnitude of effector T cell response (IFN-γ SFU per 10^6^ PBMCs) and Pseudovirus neutralization (IU/mL) against wild-type SARS-CoV-2 in prior infected, vaccinated individuals. (D) Scatter plot of magnitude of effector T cell response (IFN-γ SFU per 10^6^ PBMCs) and Pseudovirus neutralization (IU/mL) against SARS-CoV-2 Omicron variant in prior infected, vaccinated individuals. Serum neutralization of pseudotyped virus entry into ACE2-expressing 293T cells was previously reported in the same participants at the same time points (Garcia-Beltran et al., 2022). In (A–D), each dot is a single participant. Circles denote responses to wild-type peptides and squares to Omicron peptides. Dots are colored by prior infection and vaccine stratum (green for no prior infection and vaccination with primary series and orange for both prior infection and vaccination with primary series). Fill denotes sampling preboost (full filled) or postboost (half filled). Spearman correlation coefficients are denoted in each panel. Dotted lines denote a pseudoneutralization titer threshold of 20 (Garcia-Beltran et al., 2021c) and a T cell response threshold of 23.3 SFU/10^6^ PBMC (the maximal response detected among unvaccinated individuals without prior infection).

Within this cohort of individuals, we recently reported markedly reduced neutralization of Omicron following primary series vaccination, which was overcome by additional “booster” doses (Garcia-Beltran et al., 2021b). Neutralization and T cell responses against wild-type SARS-CoV-2 were correlated in magnitude in the subset of individuals with overlapping measures (Figure 4). Although additional booster doses increased both antibody and effector T cell responses, many individuals who had undetectable neutralization of Omicron pseudotyped virus had measurable T cell responses against Omicron spike prior to receipt of a booster dose. Using a pseudoneutralization titer threshold of 20 (Garcia-Beltran et al., 2022) and a T cell response threshold of 23.3 SFU/10^6^ PBMC (the maximal response detected among unvaccinated individuals without prior infection), 4/4 prior infected vaccinated individuals and 8/15 (no prior infection) vaccinated individuals who had low neutralization had measurable T cell responses. However, 38.9% (7/18) of individuals vaccinated with the primary series without prior infection demonstrated T cell reactivity and neutralization of the Omicron variant beneath the above-described threshold.

## Discussion

Immune responses induced by SARS-CoV-2 infection and/or vaccination induce a composite of antibody, effector T cell, and memory T cell responses that target viral antigens subject to mutation. In this report, we evaluated whether existing anti-SARS-CoV-2 T cell responses are cross-reactive toward the Omicron variant or differ, in comparison to wild-type and the Delta variant. We found that, in aggregate, the magnitude of circulating effector T cell responses toward Omicron spike and nonspike structural proteins was conserved across variants and enhanced by additional booster vaccine doses. However, examination of individual responses revealed that a distinct proportion of individuals with prior infection and/or vaccination have substantially reduced T cell reactivity to Omicron (but not Delta). Further evaluation of spike-specific CD4^+^ and CD8^+^ memory T cell responses revealed a significant difference in CD8^+^ T cell proliferation in response to Omicron spike relative to wild type. In sum, T cell reactivity to the SARS-CoV-2 Omicron variant was preserved in most but not all prior infected and vaccinated individuals.

The SARS-CoV-2 Omicron variant demonstrates substantial escape from neutralizing antibody responses (Garcia-Beltran et al., 2021b; Hoffmann et al., 2021) likely due to the striking enrichment of mutations at key sites in the receptor binding domain (RBD) that are critical for neutralization by antibodies (Greaney et al., 2021). In contrast, we found that T cell reactivity was relatively preserved in most individuals against Omicron, and in many individuals with undetectable Omicron neutralizing antibody responses, effector T cell responses were measurable. Conservation of sequence identity and comparable predicted HLA class I binding affinity of most putative CD8^+^ T cell epitopes between the wild-type and Omicron spike across a set of alleles with global coverage likely explains the preservation of spike-specific T cell responses observed in 80% of individuals in the study. Previous studies have identified an association between T cell immunity and mild COVID-19 disease (Peng et al., 2020; Moderbacher et al., 2020; Tan et al., 2021). In addition, in animal models of SARS-CoV-2 (McMahan et al., 2021), T cell responses appear to be important in reducing disease acquisition and severity. Thus, the high frequency of preserved T cell responses against Omicron suggests that T cell responses may be responsible for vaccine effectiveness (and also from natural infection) against severe outcomes from Omicron infection that appear higher than predicted by absent or lower neutralization.

For the subset of individuals with reduced T cell reactivity, these findings were somewhat unexpected, given that the vast majority of the spike protein in Omicron is sequence conserved in comparison to wild type (97.2%, i.e., 1,237/1,273 amino acids unchanged). However, computational HLA-peptide binding affinity assessments suggest that at least a subset of predicted unique epitopes in the Omicron spike protein may lose their ability to bind to HLA molecules. Therefore, it is possible that Omicron spike variation affects key residues critical to HLA binding that mediate escape from specific HLA-restricted T cell responses induced by prior infection and vaccination. Because HLA binding prediction algorithms are not fully reliable in their identification of immunogenic epitopes, additional experimental data by specific epitope mapping will be important to obtain. The observation that 10 study participants with reduced effector or memory T cell responses expressed between 3–6 HLA class I alleles that are putatively affected by Omicron spike mutations due to a loss in epitope binding provides additional support for this potential mechanism. Further analyses of larger cohorts of individuals could augment these findings and assist in the identification of specific HLA class I alleles that provide a disproportionate susceptibility to viral epitope escape.

Prior SARS-CoV-2 infection, despite being remote, was associated with higher T cell effector and memory responses than vaccination alone, and responses were directed against both spike and nonspike proteins in contrast to being focused solely on spike. This may reflect the impact of distinct antigen kinetics and multiple antigen exposures during infection, leading to qualitatively different responses in comparison to vaccination. The preservation of T cell reactivity to nonspike structural and accessory proteins in all individuals is likely due to the substantially reduced number of mutations within NC/M/E/3A relative to spike, suggesting that these proteins may be highly attractive for second-generation COVID-19 vaccines. In particular, vaccine strategies that induce robust memory and effector T cell responses alongside antibody responses that are collectively targeted against conserved, variant-resistant sites (Meyers et al., 2021; Nathan et al., 2021) may yield more durable T cell immunity capable of providing broad protection against future variants.

Collectively, these data provide insight into the immune mechanisms that may account for clinical observation of Omicron pathophysiology and demonstrate the contribution of vaccine boosters to enhancing cellular immunity to SARS-CoV-2 variants. They also raise the prospect that future SARS-CoV-2 variants may variably escape from antibody or T cell responses. These findings thereby support continued evaluation of second-generation vaccine approaches that induce robust T cell responses that target both variant spike and nonspike antigens in order to overcome current and future SARS-CoV-2 evolution.

### Limitations of the study

Our study has some noteworthy limitations. First, this is a study of dynamic immune responses with distinct kinetics, but timing of sampling was constrained to ∼6 months after primary series vaccination and sooner after booster doses. Second, although we included individuals with prior infection, primary series vaccination and booster vaccines with the three vaccines deployed in the USA (mRNA1273, BNT162b2, and Ad26.COV2.S), each group is relatively small and cannot comprehensively capture the variables that may plausibly affect reactivity such as the variant with which individuals were infected, the wide variety of vaccines deployed globally, differences in the timing of booster doses, and time of sampling after booster vaccination. Third, we employed IFN-γ ELISPOT and proliferation assays to estimate T cell responses. Although these assays are sensitive for functionally relevant T cell responses (Goonetilleke et al., 2006; Karlsson et al., 2003), further interrogation of the phenotype and function of responding cells could also be pursued. Moreover, additional assays to assess T cell reactivity to Omicron spike, such as the activation induced marker assay (Grifoni et al., 2020), intracellular cytokine staining following peptide stimulation, or multimer staining (Saini et al., 2021), could be employed to detect SARS-CoV-2 specific T cell responses, although a quantitative threshold of protection for any of these assays has not been clearly established. Furthermore, use of overlapping pools of 15-mer peptides in all of these assays may not fully capture the functional impact of mutations outside of antigenic epitopes in Omicron or account for the effects of viral infection on antigen processing and immune evasion (Zhang et al., 2021). Finally, we used a computational algorithm to determine the HLA restriction and binding affinity of putative epitopes in SARS-CoV-2 spike. Experimental assessment of the effects on Omicron spike mutations on individual epitopes, in terms of antigen expression, HLA binding, HLA presentation, and T cell reactivity should be pursued in future work.

## STAR★Methods

### Key resources table

**Table:** 

REAGENT or RESOURCE	SOURCE	IDENTIFIER
**Antibodies**		

Mouse monoclonal anti-human CD3 (clone OKT3)	Biolegend	Cat# 317302; RRID:AB_571927
Mouse monoclonal anti-human CD28 (clone CD28.2)	Biolegend	Cat# 302902; RRID:AB_314304
Mouse monoclonal anti-human CD3 (clone SK7) labeled with PE-Cy7 fluorophore	Biolegend	Cat# 344816; RRID:AB_10640737
Mouse monoclonal anti-human CD4 (clone RPA-T4) labeled with BV711 fluorophore	Biolegend	Cat# 300558; RRID:AB_2564393
Mouse monoclonal anti-human CD8 (clone SK1) labeled with APC fluorophore	Biolegend	Cat# 980904; RRID:AB_2564393
LIVE/DEAD Violet Viability	Life Technologies	Cat# L34960

**Biological samples**		

PBMC Specimens of prior COVID infected, vaccinated, both infected and vaccinated and booster vaccinated individuals	MGH	N/A

**Chemicals, peptides, and recombinant proteins**		

PepMix SARS-CoV-2 Wildtype Spike Peptide Pool	JPT	PM-PM-WCPV-S-1
PepMix SARS-CoV-2 Wildtype Nucleoprotein Peptide Pool	JPT	PM-WCPV-NCAP-1
PepMix SARS-CoV-2 Wildtype Membrane Peptide Pool	JPT	PM-WCPV-VME-1
PepMix SARS-CoV-2 Wildtype Envelope Peptide Pool	JPT	PM-WCPV-VEMP-1
PepMix SARS-CoV-2 Protein 3a Peptide Pool	JPT	PM-WCPV-AP3A-1
PepMix SARS-CoV-2 B.1.617.2 (Delta) Spike Peptide Pool	JPT	PM-SARS2-SMUT06-1
SARS-CoV-2 B.1.1.529 (Omicron) Spike Peptide Pool	MGH Peptide Core	N/A
SARS-CoV-2 B.1.1.529 (Omicron) NC/M/E Peptide Pool	MGH Peptide Core	N/A
CellTrace CFSE Cell Proliferation	Life Technologies	Cat# C34554

**Critical commercial assays**		

Human IFN-gamma ELISpot Basic Kit	MABTECH	Cat# 3420-2A

**Deposited data**		

Table S6	This paper	Mendeley Data: https://doi.org/10.17632/zb8vd7tpwm.1

**Software and algorithms**		

NetMHCpan – 4.1	(Reynisson et al., 2020)	https://services.healthtech.dtu.dk/service.php?NetMHCpan-4.1
Omixon HLA Explore	Omixon	N/A

### Resource availability

#### Lead contact

Further information and requests for resources and reagents should be directed to and will be fulfilled by the lead contact, Gaurav D. Gaiha (ggaiha@mgh.harvard.edu).

#### Materials availability

The overlapping pools of 15mer peptides from the Omicron spike, nucleocapsid, membrane, and envelope proteins generated in this study will be made available on request after completion of a Materials Transfer Agreement. Antibodies and other reagents are available from their respective sources.

### Experimental model and subject details

#### Human subjects

Use of human samples was approved by Massachusetts General Brigham Institutional Review Board (protocol 2020P001081). Consenting ambulatory adults in Chelsea, Massachusetts were enrolled in a study of immune responses and sampled in mid-2020 or December 2021. The median (IQR) age for the 76 donors was 47 years (36-61) with 59% (45/76) identifying as female. Additional demographic data, information regarding prior SARS-CoV-2 testing, symptoms, and exposure was collected as was vaccine related information. Prior infection was defined by positive anti-nucleocapsid antibody testing on the Roche Elecsys SARS-CoV-2 assay performed at the MGH clinical laboratory and absence of prior positive SARS-CoV-2 PCR testing. Rates of infection in the Chelsea community were high during early SARS-CoV-2 waves (Naranbhai et al., 2021) and most participants in this study had been infected in the initial waves of infection. The duration from receipt of the final dose of the primary series (first Ad26.COV2.S, or second BNT-162b2 or mRNA-1273) and duration post booster dose were collected and included as covariates as was age and sex. Samples from unvaccinated participants were obtained in 2020 (pre-Omicron period) and the remaining samples of vaccinated, prior infected and vaccinated and boosted individuals were obtained between December 3, 2021 and December 13, 2021. In total, we include 101 samples from 76 individuals; 25 individuals provided pre-booster and post-booster samples.

### Method details

#### PBMC isolation

Blood was collected in heparin tubes and processed within 4 hours of collection. Peripheral blood mononuclear cells (PBMC) were isolated by density gradient sedimentation using Lymphocyte Separation Media (Corning) as per the manufacturer’s instructions and cryopreserved in freezing media consisting of heat-inactivated fetal bovine serum (FBS, Sigma-Aldrich) containing 10% DMSO and stored in liquid nitrogen until use.

#### Peptide synthesis and analysis

Complete overlapping 15mer Spike, Nucleocapsid, Membrane, and Envelope peptides (15mer peptide overlapping by 11 amino acids) from the SARS-CoV-2 Omicron (B.1.1.529) variant were synthesized on automated robotic peptide synthesizers (AAPPTEC, 396 Models MBS, Omega and Apex) by using Fmoc solid-phase chemistry (Behrendt et al., 2016) on 2-chlorotrityl chloride resin (Barlos et al., 1991). The C-terminal amino acids were loaded using the respective Fmoc-Amino Acids in the presence of DIEA. Unreacted sites on the resin were blocked using methanol, DIEA and DCM (15:5:80 v/v). Subsequent amino acids were coupled using optimized (to generate peptides containing more than 90% of the desired full-length peptides) cycles consisting of Fmoc removal (deprotection) with 25% Piperidine in NMP followed by coupling of Fmoc-AAs using HCTU/NMM activation. Each deprotection or coupling was followed by several washes of the resin with DMF to remove excess reagents. After the peptides were assembled and the final Fmoc group removed, peptide resin was then washed with dimethylformamide, dichloromethane, and methanol three times each and air dried. Peptides were cleaved from the solid support and deprotected using odor free cocktail (TFA/triisopropyl silane/water/DODT; 94/2.5/2.5/1.0 v/v) for 2.5h at room temperature (Teixeira et al., 2002). Peptides were precipitated using cold methyl tertiary butyl ether (MTBE). The precipitate was washed 2 times in MTBE, dissolved in a solvent (0.1% trifluoroacetic acid in 30%Acetonitrile/70%water) followed by freeze drying. Peptides were characterized by Ultra Performance Liquid Chromatography (UPLC) and Matrix Assisted Laser Desorption/Ionization Mass Spectrometry (MALDI-MS). All peptides were dissolved initially in 100% DMSO at a concentration of 40 mM, prior to dilution at the appropriate concentration to create protein-specific peptide pools in RPMI-1640 medium.

#### SARS-CoV-2 antigens

Peptide pools of 15mer sequences (overlapping by 11 amino acids) covering the full length of wildtype spike, nucleocapsid, membrane, envelope and ORF3A were obtained from a commercial source (JPT peptide technologies). Overlapping Delta spike peptide pools were also obtained from JPT. For Omicron spike, nucleocapsid, membrane and envelope peptide pools, 15mer peptides (overlapping by 11 amino acids) covering the full length of the mutated proteins were individually synthesized as crude material (MGH Peptide Core). All peptides were individually resuspended in dimethyl sulfoxide (DMSO) at a concentration of 40 mg/mL. Peptide pools for Omicron spike and non-spike proteins were created by pooling aliquots of individual peptides and resuspension in RPMI and DMSO at 20 μg/mL. Pools were used at a final concentration of 0.25-0.5 μg/mL with an equimolar DMSO concentration in the non-stimulated control.

#### *Ex vivo* IFN-γ ELISPOT

IFN-γ ELISpot assays were performed according to the manufacturer’s instructions (Mabtech). PBMCs (1-2x10^5^/well) were incubated with SARS-CoV-2 peptide pools at a final concentration of 0.5 μg/ ml for 16–18h. Anti-CD3 (Clone OKT3, Biolegend, 1ug/mL) and anti-CD28 Ab (Clone CD28.2, Biolegend, 1ug/mL) were used as positive controls. To quantify antigen-specific responses, mean spots of the DMSO control wells were subtracted from the positive wells, and the results were expressed as spot-forming units (SFU) per 10^6^ PBMCs. Responses were considered positive if the results were >5 SFU/10^6^ PBMCs following control subtraction. If negative DMSO control wells had >30 SFU/10^6^ PBMCs or if positive control wells (anti-CD3/anti-CD28 stimulation) were negative, the results were excluded from further analysis. For graphical analyses, negative responses are plotted at a value of 1 SFU/10^6^ PBMCs.

#### CFSE proliferation assay

PBMCs were suspended at 1 x 10^6^/mL in PBS and incubated at 37^°^C for 20 min with 0.5 uM carboxyfluorescein succinimidyl ester (CFSE; Life Technologies). After the addition of serum and washes with PBS, cells were resuspended at 1 x 10^6^/mL and plated into 96-well U-bottom plates (Corning) at 200 uL volumes. Peptide pools were added at a final concentration of 0.25 ug/mL. On day 6, cells were harvested, washed with PBS + 2% Fetal Bovine Serum, and stained with anti-CD3-PE-Cy7 (clone SK7; BioLegend), anti-CD8 APC (clone SK1; BioLegend), anti-CD4 BV711 (clone RPA-T4; BioLegend) and LIVE/DEAD violet viability dye (Life Technologies). Cells were washed and fixed in 2% paraformaldehyde, prior to flow cytometric analysis on a BD LSR II (BD Biosciences). A positive response was defined as one with a percentage of CD3^+^ CD8^+^ or CD3^+^ CD4^+^ CFSE low cells at least 1.5x greater than the highest of two negative-control wells and greater than 0.2% CD8^+^ or CD4^+^ CFSE low cells in magnitude following background subtraction. For graphical analyses, responses are plotted at a value of 0.1% CD8^+^ or CD4^+^ CFSE low cells.

#### HLA binding affinity analysis of predicted CD8^+^ T-cell epitopes

Predicted binding affinities for all 8-11mer peptides in wildtype spike (N = 5058) and Omicron spike (N = 5050) to 150 HLA-A, -B, and -C alleles were calculated using NetMHCpan4.1 (https://services.healthtech.dtu.dk/service.php?NetMHCpan-4.1). Strong binders were those with an EL-Rank score <0.5, while weak binders had scores >0.5 and <2.0 and non-binders had scores >2.0, with thresholds set as defined by NetMHC.

#### HLA typing

Locus-specific PCR primers were used to amplify polymorphic exons of *HLA-A, HLA-B, HLA-C* genes with the Fluidigm Access Array (Fluidigm). PCR amplicons were pooled and sequenced on an Illumina MiSeq platform (Illumina). *HLA* alleles and genotypes were called using the Omixon HLA Explore (beta version) software (Omixon). Ambiguous calls were resolved by Sanger sequencing.

#### Neutralization of wildtype and Omicron Spike pseudotyped virus

Neutralization data is from our recent study in a subset of individuals described here and previously reported (Garcia-Beltran et al., 2021b). In brief, pseudovirus neutralization titer 50 (pNT50) was calculated by taking the inverse of the serum concentration that achieved 50% neutralization of SARS-CoV-2 pseudotyped lentivirus particles entry into ACE2 expressing 293T cells (a gift from Michael Farzan). We introduced mutations corresponding to the SARS-CoV-2 variants of concern by site directed mutagenesis and confirmed clones by sequencing.

### Quantification and statistical analysis

The primary statistical analysis shown in Tables S3 and S5 was a multivariate regression modelling T-cell response (log_10_ CFU/10^6^ PBMC) as the response variable, and age, sex, peptide pool, prior infection, vaccine type, duration from vaccination as covariates. To compare proportions of individuals we used a fishers-exact test. Analyses were performed in R and figures rendered in GraphPad Prism. A p-value of < 0.05 was considered significant.

## Data Availability

• Full list of predicted HLA-restricted spike epitopes and their binding affinities is available from Mendeley Data: https://doi.org/10.17632/zb8vd7tpwm.1. All other data supporting the findings of this study are available from the lead contact upon request. • This paper does not report original code. • Any additional information required to reanalyze the data reported in this work is available from the lead contact upon request.

## Supplemental information

Document S1. Tables S1–S5 and S7

Table S6. CD8^+^ T cell epitopes in the SARS-CoV-2 spike protein (736 unique epitopes) predicted to bind HLA alleles, related to Table 1
