# T cell reactivity to the SARS-CoV-2 Omicron variant is preserved in most but not all individuals

**DOI:** 10.1016/j.cell.2022.03.022

**Published:** 2022-03-31

**Authors:** Vivek Naranbhai, Anusha Nathan, Clarety Kaseke, Cristhian Berrios, Ashok Khatri, Shawn Choi, Matthew A. Getz, Rhoda Tano-Menka, Onosereme Ofoman, Alton Gayton, Fernando Senjobe, Zezhou Zhao, Kerri J. St Denis, Evan C. Lam, Mary Carrington, Wilfredo F. Garcia-Beltran, Alejandro B. Balazs, Bruce D. Walker, A. John Iafrate, Gaurav D. Gaiha

(Cell *185*, 1041–1051.e1–e6; March 17, 2022)

In our originally published article, an error in production resulted in mistakes to the citations and references for Garcia-Beltran et al., 2021a, 2021b, and 2022. At publication, Garcia-Beltran et al., 2021a referred to the Correction for the original Garcia-Beltran et al., 2021a, which ran in *Cell* volume 184 on page 2532; instead, it should refer to the original article, which ran in *Cell* volume 184 on pages 2372–2383.e9. Garcia-Beltran et al., 2021b referred to the BioRxiv pre-print publication of Garcia-Beltran et al., 2022; instead, it should refer to Garcia-Beltran et al., 2021, Cell *184*, 476–488.e11. The references have now been corrected online.

To coincide with corrections to the references, the citations in the following sentences have also been corrected as indicated:

• “Additional booster vaccine doses partially compensate for this effect (Garcia-Beltran et al., 2021b; Hoffmann et al., 2021)” now refers to Garcia-Beltran et al. (2022).

• “Within this cohort of individuals, we recently reported markedly reduced neutralization of Omicron following primary series vaccination, which was overcome by additional ‘booster’ doses (Garcia-Beltran et al., 2021b)” now refers to Garcia-Beltran et al. (2022).

• “Using a pseudoneutralization titer threshold of 20 (Garcia-Beltran et al., 2022)” now refers to Garcia-Beltran et al. (2021b).

• In Figure 4D, “Dotted lines denote a pseudoneutralization titer threshold of 20 (Garcia-Beltran et al., 2021c)” now refers to Garcia-Beltran et al. (2021b).

• “The SARS-CoV-2 Omicron variant demonstrates substantial escape from neutralizing antibody responses (Garcia-Beltran et al., 2021b; Hoffmann et al., 2021)” now refers to Garcia-Beltran et al. (2022).

The journal apologizes for any confusion these errors may have caused.

