# Supplementary material for: T cell reactivity to the SARS-CoV-2 Omicron variant is preserved in most but not all individuals
Source: Cell. 2022 Mar 17;185(6):1041–1051.e6. doi: 10.1016/j.cell.2022.01.029 (PMC8810349; doi:10.1016/j.cell.2022.01.029)
Supplement: Document S1. Tables S1–S5 and S7 [file mmc1.pdf]

Cell, Volume *185*

## Supplemental information

# T cell reactivity to the SARS-CoV-2 Omicron variant is preserved in most but not all individuals

**Vivek Naranbhai, Anusha Nathan, Clarety Kaseke, Cristhian Berrios, Ashok Khatri, Shawn Choi, Matthew A. Getz, Rhoda Tano-Menka, Onosereme Ofoman, Alton Gayton, Fernando Senjobe, Zezhou Zhao, Kerri J. St Denis, Evan C. Lam, Mary Carrington, Wilfredo F. Garcia-Beltran, Alejandro B. Balazs, Bruce D. Walker, A. John Iafrate, and Gaurav D. Gaiha**

**Table S1: Baseline characteristics of included samples (N=101 from 76 unique donors), related to Figure 1.**

| | Unvaccinated, N = 21 | pre-boost, N = 36# | post-boost, N = 44# |
|---|---|---|---|
| **Age** | 34 (28, 58) | 46 (38, 54) | 48 (40, 64) |
| **Sex** | | | |
| Female | 9 (43%) | 26 (72%) | 30 (68%) |
| Male | 12 (57%) | 10 (28%) | 14 (32%) |
| **Prior infection** | | | |
| PCR/Antibody confirmed prior infection | 11 (52%) | 12 (33%) | 13 (30%) |
| No prior infection | 10 (48%) | 24 (67%) | 31 (70%) |
| **Vaccine type** | | | |
| None | 21 (100%) | 0 (0%) | 0 (0%) |
| Ad26.COV2.S | NA | 4 (11%) | 7* (16%) |
| BNT-162b2 | NA | 24 (67%) | 27** (61%) |
| mRNA-1273 | NA | 8 (22%) | 10*** (23%) |
| **Duration after primary vaccination series (median days (IQR))** | NA | 220 (207, 243) | 232 (216, 250) |
| **Booster type** | | | |
| heterologous | NA | NA | 7 (16%) |
| homologous | NA | NA | 37 (84%) |
| **Duration after booster (median days (IQR))** | NA | NA | 10 (9, 15) |

# includes paired paired pre-boost and post-boost samples from 25 individuals

* includes 3 boosted with Ad26.COV2.S, 2 with mRNA-1273 and 2 with BNT162b2

** includes 25 boosted with BNT162b2 and 2 with mRNA-1273

**Table S2: Details of variant peptide pools for spike and non-spike structural proteins, related to Figure 1.**

| | Spike | | | Nucleocapsid, Membrane, Envelope, ORF3A | |
|---|---|---|---|---|---|
| | Wildtype | Delta | Omicron | Wildtype | Omicron |
| **Number of peptides in pool** | 315 | 315 | 315 | 237 | 237 |
| **Peptides shared with wildtype** | Ref | 288 (91.4%) | 229 (72.7%) | Ref | 213 (89.9%) |
| **Peptides unique to variant** | Ref | 27 (8.6%) | 86 (27.3%) | Ref | 24 (10.1%) |

**Table S3: Multivariate regression of total T-cell response to SARS-CoV-2 spike peptides in vaccinated individuals, related to Figures 1 and 2.**

| | Effect estimate on total T-cell response (in $\log_{10}$ CFU/$10^6$ PBMC) | 95% Confidence Interval | p-value |
|---|---|---|---|
| **Age (per 10 year increase)** | -0.03 | -0.09, 0.03 | 0.3 |
| **Sex** | | | |
| Female | Ref | | |
| Male | -0.03 | -0.21, 0.15 | 0.7 |
| **SARS-CoV-2 variant** | | | |
| ancestral | Ref | | |
| Delta (B.1.617.2) | -0.09 | -0.27, 0.09 | 0.3 |
| Omicron (B.1.1529) | -0.03 | -0.21, 0.15 | 0.7 |
| **Prior infection** | | | |
| No prior infection | Ref | | |
| PCR/Antibody confirmed prior infection | 0.55 | 0.38, 0.72 | <0.001 |
| **Vaccine type** | | | |
| mRNA-1273 | Ref | | |
| BNT-162b2 | 0.09 | -0.10, 0.28 | 0.3 |
| Ad26.COV2.S | -0.05 | -0.32, 0.22 | 0.7 |
| **Duration after primary vaccination series in weeks** | -0.02 | -0.05, 0.00 | 0.028 |
| **Booster group** | | | |
| pre-boost | Ref | | |
| post-boost | 1.1 | 0.91, 1.2 | <0.001 |

**Table S4. Frequency of individuals with greater than 50% (0.3$\log_{10}$) reduction in in circulating effector T-cell response to variant, relative to wildtype, related to Figures 1 and 2.**

| | | Unvaccinated | Pre-booster | Post-booster | Overall |
|---|---|---|---|---|---|
| **Omicron** | **Prior infected** | 27.3% (3/11) | 8.3% (1/12) | 15.4% (2/13) | 15.3% (14/91)* |
| | **No prior infection** | - | 25% (6/24) | 6.5% (2/31) | |
| **Delta** | **Prior infected** | 18.2% (2/11) | 0% (0/12) | 0% (0/13) | 5.9% (5/85) |
| | **No prior infection** | - | 16.7% (3/18) | 0% (0/31) | |

*Fisher's exact test comparing frequency for Omicron vs. Delta in 85 individuals with both measured, i.e. 13/85 5/85; p-value 0.024.

**Table S5: Multivariate regression of $CD4^+$ and $CD8^+$ T-cell proliferative response to SARS-CoV-2 spike peptides in vaccinated individuals, related to Figures 1 and 2.**

| | CD4+ Memory T cell response | | | CD8+ Memory T cell response | | |
|---|---|---|---|---|---|---|
| | Effect estimate | 95% Confidence Interval | p-value | Effect estimate | 95% Confidence Interval | p-value |
| **Age (per 10 year increase)** | 0.16 | -1.2, 1.5 | 0.8 | -0.67 | -2.2, 0.88 | 0.4 |
| **Sex** | | | | | | |
| Female | Ref | | | | | |
| Male | 0.72 | -3.1, 4.5 | 0.7 | 0.72 | -3.1, 4.5 | 0.7 |
| **SARS-CoV-2 variant** | | | | | | |
| ancestral | Ref | | | | | |
| Omicron (B.1.1529) | -1 | -4.5, 2.5 | 0.6 | -3.4 | -7.4, 0.58 | 0.093 |
| **Prior infection** | | | | | | |
| No prior infection | Ref | | | | | |
| PCR/Antibody confirmed prior infection | 0.42 | -3.2, 4.1 | 0.8 | 3.6 | -0.54, 7.8 | 0.087 |
| **Vaccine type** | | | | | | |
| mRNA-1273 | Ref | | | 4.1 | -0.79, 9.0 | 0.1 |
| BNT-162b2 | 1.6 | -2.7, 5.9 | 0.5 | 0.28 | -9.0, 9.6 | >0.9 |
| Ad26.COV2.S | 2 | -6.2, 10 | 0.6 | | | |
| **Duration after primary vaccination series in weeks** | -0.28 | -0.74, 0.18 | 0.2 | -0.01 | -0.53, 0.51 | >0.9 |
| **Booster group** | | | | | | |
| pre-boost | Ref | | | | | |
| post-boost | 3.2 | -1.2, 7.5 | 0.2 | 5.4 | 0.48, 10 | 0.032 |

**Table S7: HLA genotypes of representative participants who demonstrated >50% decline in T-cell reactivity to SARS-CoV-2 Omicron, related to Table 1.**

| Participant | HLA-A Allele 1 | HLA-A Allele 2 | HLA-B Allele 1 | HLA-B Allele 2 | HLA-C Allele 1 | HLA-C Allele 2 | Number of HLA-I alleles predicted to be affected by loss in Omicron binding |
|---|---|---|---|---|---|---|---|
| 1 | **11:01** | **30:01** | **15:01** | **42:02** | **01:02** | 17:01 | 5 |
| 2 | **01:01** | **29:02** | **08:01** | 44:03 | 07:01 | **16:01** | 4 |
| 3 | 02:01 | **26:01** | **08:01** | **38:01** | 07:01 | **12:03** | 4 |
| 4 | **23:01** | 80:01 | 18:01 | 44:03 | **02:02** | **04:01** | 3 |
| 5 | 02:01 | **32:01** | **35:01** | 58:01 | **04:01** | 07:01 | 3 |
| 6 | **24:02** | **24:02** | **15:20** | **15:20** | **01:02** | **01:02** | 6 |
| 7 | **01:01** | **68:01** | 50:02 | **52:01** | **06:02** | **12:02** | 5 |
| 8 | 02:01 | **29:02** | **35:01** | 51:01 | **04:01** | **15:09** | 4 |
| 9 | 31:01 | 68:03 | **39:05** | **40:02** | 03:05 | **07:02** | 3 |
| 10 | **23:01** | **30:02** | 40:01 | 51:01 | 03:04 | **15:02** | 3 |

*Bolded HLA class I alleles are those where binding to wildtype epitopes is affected by mutations in Omicron.*
